# Predicting Implantation Outcome of *In Vitro* Fertilization and Intracytoplasmic Sperm Injection Using Data Mining Techniques

**DOI:** 10.22074/ijfs.2017.4882

**Published:** 2017-09-03

**Authors:** Pegah Hafiz, Mohtaram Nematollahi, Reza Boostani, Bahia Namavar Jahromi

**Affiliations:** 1 Department of Medical Informatics, School of Management and Medical Informatics, Shiraz University of Medical Sciences, Shiraz, Iran; 2 Anesthesiology and Critical Care Research Center, Shiraz University of Medical Sciences, Shiraz, Iran; 3 Department of Computer Science and Engineering and Information Technology, School of Electrical and Computer Engineering, Shiraz University, Shiraz, Iran; 4 Department of Obstetrics and Gynecology, School of Medicine, Shiraz University of Medical Sciences, Shiraz, Iran; 5 Infertility Research Center, Shiraz University of Medical Sciences, Shiraz, Iran

**Keywords:** *In Vitro* Fertilization, Intracytoplasmic Sperm Injection, Clinical Decision Support, Data Mining

## Abstract

**Background:**

*In vitro* fertilization (IVF) and intracytoplasmic sperm injection (ICSI) are two important subsets of the assisted reproductive techniques, used for the treatment of infertility. Predicting implantation outcome of IVF/ICSI or the chance of pregnancy is essential for infertile couples, since these treatments are complex and expensive with a low probability of conception.

**Materials and Methods:**

In this cross-sectional study, the data of 486 patients were collected using census method. The IVF/ICSI dataset contains 29 variables along with an identifier for each patient that is either negative or positive. Mean accuracy and mean area under the receiver operating characteristic (ROC) curve are calculated for the classifiers. Sensitivity, specificity, positive and negative predictive values, and likelihood ratios of classifiers are employed as indicators of performance. The state-of-art classifiers which are candidates for this study include support vector machines, recursive partitioning (RPART), random forest (RF), adaptive boosting, and one-nearest neighbor.

**Results:**

RF and RPART outperform the other comparable methods. The results revealed the areas under the ROC curve (AUC) as 84.23 and 82.05%, respectively. The importance of IVF/ICSI features was extracted from the output of RPART. Our findings demonstrate that the probability of pregnancy is low for women aged above 38.

**Conclusion:**

Classifiers RF and RPART are better at predicting IVF/ICSI cases compared to other decision makers that were tested in our study. Elicited decision rules of RPART determine useful predictive features of IVF/ICSI. Out of 20 factors, the age of woman, number of developed embryos, and serum estradiol level on the day of human chorionic gonadotropin administration are the three best features for such prediction.

## Introduction

Assisted reproductive technologies (ART) include all treatments that are used for *in vitro* handling of human oocytes and sperms or of the embryos to establish a pregnancy (1). Infertility is defined as a couple’s inability to conceive after 12 months of regular unprotected intercourse (2). Among ART treatments, *In vitro* fertilization (IVF) and intracytoplasmic sperm injection (ICSI) are well-known methods for infertility treatment. The process of IVF involves ovarian stimulation, oocyte retrieval, fertilization, embryo culture, and transferring embryos to the uterus (3). ICSI is another treatment used for infertile couples that includes injection of a selected sperm into the oocyte cytoplasm (4).

IVF and ICSI have almost similar variables in terms of demographical and clinical features. The latest study in Iran (5) demonstrates that the total average rate of infertility is about 10.9% of the population. This study states that among patients of several infertility clinics in the country, 78.4% had primary and 21.6% had secondary fertility factors. The results yield 34.0% of the average percentage for male factor, 43.5% for female factor, 17.1% for both factors, and 8.1% for unexplained infertility. Ovulatory dysfunction was the most frequent etiologic factor among female causes in that study.

Today, many couples suffering from infertility try ART to have a baby and ask about the probability of pregnancy due to several reasons. Firstly, due to the high cost of IVF and ICSI treatments in Iran, some couples cannot afford the cost of these treatments. Next, the probability of conception is 20 to 25% in a normal reproductive cycle (3), which by ART increases to about 30-40% in each cycle; however, it is still considered to be low. Lastly, ART consists of multiple steps that are time consuming and difficult to tolerate by infertile women. There are also three main clinical causes that make predicting pregnancy outcome necessary. First, there are many prognostic factors to this treatment that determine the chance of conceiving, which in turn make the decision difficult for clinicians. Second, using previous cases for this decision seems to be reliable, while it is a time-consuming task for clinicians. And last, there might be an alternative method to IVF and ICSI that a specialist proposes to couples with a very low chance of pregnancy, such as adoption, that causes them to call off infertility treatments. Data mining (DM) refers to using machine learning, pattern recognition, and statistical techniques to extract knowledge from data, in this case, patient information, and is a specific step in the process of knowledge discovery in databases (KDD) (6). In medical DM, classification system predicts the class to which the patient belongs by learning a model based on input dataset. Since DM methods perform data analysis and elicit valuable information from data, clinical obstetricians and gynecologists may use such information for diagnosis and treatment (7). According to Cios and Moore (8), medical DM can be beneficial for patients when finding a solution to analyze various types of clinical data.

In this study, five well-known classification techniques in DM are applied to our dataset along with 5-fold cross validation (CV) for training and testing. The main purpose of this research was to choose the best predictive model for calculating the probability of IVF/ICSI success for each couple, using a comparative study among various classifiers. Furthermore, we aimed to find the most effective factors for prediction of ART success in infertile couples. Note that classical predictive models could be used in this study; however, the methods used here are limited to DM approach to examine the effectiveness of artificial intelligence on the subject. In addition, DM discovers patterns from data and considers computational efficiency comparing to classical predictive models. There are several studies performed to predict IVF outcomes (9-12), where different methods have been used to predict IVF success with accuracies from 60.6% (9) to 84.4% (11).

In another similar study, unlike the attempts that solely consider accuracy, Güvenir et al. (11) utilized the area under ROC curve (AUC) as the performance criterion since it is practical in evaluating quality of the algorithm. Our dataset has 17 variables in common with the study of Guh et al. (10). Some of the features, like the information about the first and second stage culture medias, were not documented in our infertility center. In the study of Güvenir et al. (11), 19 variables similar to our database were used. Some of the variables such as anemia, which were used in their study were not considered as predictive features of IVF/ICSI by our infertility specialist as predictive features of IVF/ICSI, and therefore, were not used in our study. Finally, another similar study conducted by Chen et al. (12) used 9 variables in common with our dataset. The only variable that our infertility specialists considered a significant predictive feature, which was not seen in previous studies, was the number of gonadotropin ampules that were used for our patients.

## Materials and Methods

A dataset of 486 labeled records along with 29 variables was gathered belonging to Infertility Research Center of Mother-and-Child Hospital in Shiraz, Iran, from 2009 to 2015. Each patient signed a consent form at the time of admission to the hospital and before entering the study. This study was approved by Ethics Committee of Shiraz University of Medical Sciences. The type of this study is cross-sectional and the method of sampling is census. This dataset contained 131 positive and 355 negative implantations. As far as the number of negative samples outnumbers positive ones, this dataset is highly imbalanced. Required variables for this study were extracted from paper-based medical records by our trained staff. In order to use these records for computer models, data entry process was performed. In this study, frozen embryo implantation results were excluded and only fresh embryo transfer was considered due to the diversity of some features between these two transferring methods. The name, type, and value of IVF/ICSI attributes are summarized (Table 1).

**Table 1:** IVF/ICSI attributes of our dataset


Attribute name	Attribute type	Attribute value

Age of woman	Numeric	18-47
Age of man	Numeric	23-70
Body mass index	Numeric	14.53-45.78
Secondary fertility	Text	Yes, no
Tubal factor	Text	Yes, no
Pelvic factor	Text	Yes, no
Ovulatory factor	Text	Yes, no
Uterine factor	Text	Yes, no
Male factor	Text	Yes, no
Infertility duration	Numeric	1-27
Experience of IVF treatment	Text	Yes, no
Sperm count	Numeric	0-513 (in million)
Sperm morphology	Numeric	0-95%
Sperm motility	Numeric	0-85%
Follicle stimulating hormone	Numeric	0.099-51.7
Anti-mullerian hormone	Numeric	0.01-93.93
Antral follicle counts	Numeric	2-57
Number of gonadotropin ampoules	Numeric	8-110 (in 75 units)
Number of follicles in ultrasound	Numeric	1-35
Serum E2 level on the day of hCG administration	Numeric	0.95-32840.8
Number of retrieved oocytes	Numeric	0-44
Number of oocytes of GV quality	Numeric	0-8
Number of oocytes of MI quality	Numeric	0-8
Number of oocytes of MII quality	Numeric	0-27
Type of treatment	Text	IVF, ICSI
Embryo grade	Text	A, B, C, D
Number of developed embryos	Numeric	0-26
Embryo transfer day	Numeric	2,3,4
Number of transferred embryos	Numeric	0-6


IVF; *In vitro* fertilization, ICSI; Intracytoplasmic sperm injection, hCG; Human chorionic gonadotropin, E2; Estradiol, GV; Germinal vesicle, MI; Metaphase I, and MII; Metaphase II.

Preparation of raw data is one of the most important steps in knowledge discovery. The importance of data preparation is discussed by Zhang et al. (13). This study asserts that almost 80% of the total efforts were spent on preparing data. The patients’ records had missing values in some features; therefore, the power of classifiers declined in some cases. The most common methods in literature are case deletion, mean imputation, median imputation, and k-nearest neighbor (kNN) imputation (14).

Since the attributes with missing values in our dataset had skewed distribution, the missing values of numerical features are replaced with median and categorical attributes are filled with the mode of their corresponding column. Support vector machines (SVM), recursive partitioning (RPART), random forest (RF), Adaptive boosting (Adaboost), and 1NN are the stateof- art techniques employed in this research for intelligent decision making. These models are compared to each other for choosing the best option in order to predict IVF/ ICSI, as well as obtaining the probability of each decision rule. For implementation of the mentioned classifiers, we used R 3.2.3. and a five-fold stratified CV is utilized for the validation phase. K-fold CV (15) is a common technique for performance evaluation which reports the average output for classifiers. Since ROC is a good criterion for imbalance datasets, the AUC of ROC is selected as the performance measure instead of accuracy. Visualization of ROC curves is used frequently as performance graphing approach in medical decision making (16). Finally, sensitivity, specificity, positive predictive values (PPV), negative predictive values (NPV), positive likelihood ratio (LR+), and negative likelihood ratio (LR) are also calculated (17).

## Results

We applied the processed samples to each classifier to calculate AUC and accuracy over 5-fold CV, and represented them as mean values (Table 2). Each experiment is repeated 20 times to examine a comprehensive combination of data samples. The average over these experiments for each classifier is reported besides standard deviation. In addition, specificity, sensitivity, PPV, NPV, LR+, and LR- are also calculated for each classifier (Table 3). Our findings suggest that RF and RPART outperform other classifiers in terms of specificity, PPV, and NPV. RPART predicts positive cases better than RF; however, negative cases are classified by RF better than RPART. The higher value of PPV in RF is due to the lower number of false positives. Seemingly, the higher number of NPV in RPART is because of the lower number of false negatives in confusion matrices of both models. Adaboost has generally better values especially in terms of sensitivity comparing to SVM, and 1NN. While the specificity of SVM is 88.73% and higher than 1NN, its value for specificity (14.5%) is very low. Interestingly, given a positive pregnancy, the high positive likelihood ratio of RF shows a large increase in the likelihood of pregnancy, and the corresponding value for RPART implies a moderate increase. However, the rest of the models result in minimal increases. The negative likelihood ratios of all classifiers, which are almost between 0.5 and 1, represent minimal decrease in the probability of pregnancy.

**Table 2:** Experimental results of applying SVM, Adaboost, RPART, RF, and 1NN on our dataset. All values are rounded to two digitis after the decimal


	AUC (%)	Accuracy (%)

SVM	57.57 ± 1.51	68.3 ± 1.05
Adaboost	47.52 ± 4.5	66.99 ± 2.85
RPART	82.05 ± 2.34	83.56 ± 0.99
RF	84.23 ± 0.91	83.96 ± 0.62
1NN	50 ± 0	64.84 ± 1.46


SVM; Support vector machines, RPART; Recursive partitioning, RF; Random forest, 1NN; One-Nearest-Neighbor, Adaboost; Adaptive boosting, and AUC; Areas under the ROC curve.

**Table 3:** Sensitivity, specificity, PPV, NPV, LR+, and LR- of RF, RPART, Adaboost, SVM and 1NN for models. All values are rounded to two digits after the decimal


	Sensitivity (%)	Specificity (%)	PPV (%)	NPV (%)	LR+	LR-

RF	48.85	98.03	90.14	83.86	24.78	0.52
RPART	59.54	91.83	72.90	86.02	7.29	0.44
Adaboost	54.96	70.42	40.68	80.91	1.86	0.64
SVM	14.5	88.73	32.20	73.77	1.29	0.96
1NN	35.88	73.52	33.33	75.65	1.35	0.87


PPV; Positive predictive values, NPV; Negative predictive values, LR+; Positive likelihood ratio, LR-; Negative likelihood ratio, RF; Random forest, RPART; Recursive partitioning, SVM; Support vector machines, 1NN; One-Nearest-Neighbor, and Adaboost; Adaptive boosting.

**Fig.1:**
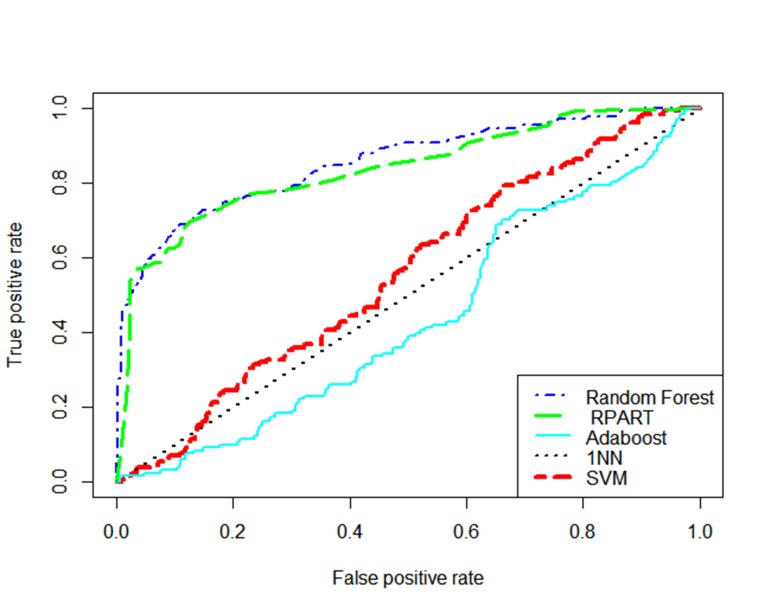
Receiver operating characteristic curves of all classifiers.

Among all tested classifiers in this study, RPART leads to the most usable information besides the probability of IVF/ICSI success. Therefore, we present the significance of the 20 features of IVF/ICSI using RPART (Table 4). The second column shows the scores of each feature. Note that only 11 features have specific values for positive pregnancy because these features were significant in RPART decision making. The other 9 variables are not considered in predicting IVF/ICSI outcome, as they did not have specific values for positive pregnancy. Figure 1 shows ROC curves for predictive models, using all of the data samples. As it is apparent, RF and RPART have higher AUC comparing to Adaboost, SVM, and 1NN, and the curve of SVM is closer to the top two classifiers than 1NN and Adaboost.

**Table 4:** Importance of IVF/ICSI variables using RPART


Variable	Score	Values for positive pregnancy

Age of woman	14	<38
Number of developed embryos	13	>3 and <16
Serum E2 level	12	<1040 and ≥1780
Embryo grade	9	A, B and C
Sperm motility	9	≥62%
Type of treatment	5	ICSI
Sperm count	5	>4.5 million
Embryo transfer day	4	3 and 4 days
AFC	4	<10
Infertility duration	3	<7.5 years
AMH	3	≥1.2
Number of transferred embryos	3	Not specific
Number of retrieved oocytes	3	Not specific
Number of Gonadotropin ampules	3	Not specific
Sperm morphology	3	Not specific
FSH	2	Not specific
Male factor	2	Not specific
Age of man	1	Not specific
Number of follicles	1	Not specific
Ovulatory factor	1	Not specific


IVF; *In vitro* fertilization, ICSI; Intracytoplasmic sperm injection, RPART; Recursive partitioning, E2; Estradiol, AFC; Antral follicle counts, AMH; Anti- Mullerian hormone, and FSH; Follicle stimulating hormone.

## Discussion

DM methods used in this research involved a learning process, which utilizes previous IVF/ ICSI records to predict the outcome of a new test case. This property improves the decision making of the physicians using previous cases. The low probability of success for a test case obtained by applying DM methods is practical for domain experts to prevent couples from choosing IVF/ICSI treatments. SVM, on the other hand, is suitable for binary classification tasks. It has been employed in many artificial intelligence fields, such as medical diagnosis. Since medical datasets are naturally imbalanced, SVM boundary will be biased in favor of the class with higher population, hence unsatisfactory results of SVM model obtained in this experiment are expected. KNN is a simple nonparametric distance-based method used in many applications. The complexity of kNN is highly dependent on the number of attributes and instances (18). In a study by Japkowicz and Stephen (19) the low performance of kNN when facing imbalanced dataset is demonstrated. Furthermore, kNN performance can be declined in noisy environments since the neighbors of each input take the decision about its label.

Although Adaboost is a strong ensemble learner that can construct a flexible boundary between the classes, it highly suffers from high sensitivity to noisy samples. This deficiency is due to the learning process of Adaboost in which learning of weak learners is performed sequentially; therefore, outlier and noisy samples are boosted in successive iterations and make the learners highly biased to these samples. The set of IVF/ICSI predictive features in our findings indicates that the age of a women who is seeking IVF/ICSI treatment, plays the most important role in making a decision whether to proceed with thesetreatments. Features with the same score are considered to be equally significant, like infertility duration and anti-Mullerian hormone (AMH) testing features. In a study done by Lintsen et al. (20), they claimed that age of a woman is the key feature in the success of IVF/ICSI and those with the age of over 35 had a lower chance of pregnancy. The threshold obtained by the decision tree method is determined 38 years old. Another interesting finding is that AMH and antral follicle count features, which have close scores to each other, are considered to be accurate in predicting excessive response of ovarian hyperstimulation in IVF/ICSI treatment (21).

It has been previously demonstrated that AUC performs better than the accuracy index for comparing different learning algorithms (22, 23). Among former investigations, only Güvenir et al. (11) considered AUC as the main criterion. The mean AUC obtained in their study was 83.3%, which is close to the values obtained from RF and RPART in our study. The age of a woman is also indicated as the most remarkable feature for two out of three methods employed in the studies by Guh et al. (10); however, the set of features in their dataset differs from our dataset. One of the major limitations of this work was the number of IVF/ ICSI records. This problem was mainly due to the number of incomplete patients’ records available to us. In addition, the newly-established center from which our dataset was gathered didn’t have enough considerable records of patients who did fresh embryo transfers. The other problem was missing values that affected the power of classifiers, since missing values decrease the accuracy of the classifiers. This issue affects the values of ranked features, providing positive value for pregnancy.

A restriction of the current study is that classical predictive models like Templeton, logistic regression, and Bayesian method are not considered for comparison since the focus of this study was only on a set of DM techniques. Note that logistic regression, for example, has a major limitation, which is the features of a dataset should be independent from each other. For example, follicle-stimulating hormone (FSH) and AMH are two features that have inverse relationship with each other. Also, a woman’s age has proved correlations with AMH, FSH, the number of oocytes, and embryo quality. Nevertheless, in order to obtain a more comprehensive comparison, classical predictive models should have been used besides the DM models obtained in this study. Further studies should develop a suitable algorithm to tackle the problem of class imbalance for the classifiers that are sensitive to dissimilarity of the distribution of the classes. Ideally, it would be very helpful for such predictive analyses if healthcare institutes around the world would design a global database for IVF and ICSI, or ART in general. In that case, the results would be more generalized and comparable to each other. Presently, the variability in ART success among research centers provides different or in some cases contradictory results, which cannot be ignored.

## Conclusion

According to the obtained results in the current study, RF and RPART outperformed the other methods for pregnancy prediction with AUC of 84.23 and 82.05%, respectively. Besides the issue of classifiers, knowledge in the form of selected features is extracted from RPART model. Age of a woman, number of developed embryos, and serum estradiol (E2) level on the day of human chorionic gonadotropin (hCG) administration are introduced as the best three predictive features for IVF/ICSI.
